# If you don’t test, they will not treat: Impact of stopping preoperative screening for asymptomatic bacteriuria

**DOI:** 10.1017/ash.2023.166

**Published:** 2023-05-26

**Authors:** Marisa L. Winkler, Joanne Huang, Jessica Starr, David C. Hooper, Molly L. Paras, Alyssa R. Letourneau, Erica S. Shenoy

**Affiliations:** 1 Division of Infectious Disease, Massachusetts General Hospital, Boston, Massachusetts; 2 Department of Medicine, Harvard Medical School, Boston, Massachusetts; 3 Department of Microbiology, Brigham and Women’s Hospital, Boston, Massachusetts; 4 Infection Control Unit, Massachusetts General Hospital, Boston, Massachusetts; 5 Department of Pharmacy, Massachusetts General Hospital, Boston, Massachusetts; 6 Department of Cardiac Surgery, Massachusetts General Hospital, Boston, Massachusetts

## Abstract

**Objective:**

Screening for asymptomatic bacteriuria (ASB) is not recommended outside of patients undergoing invasive urological procedures and during pregnancy. Despite national guidelines recommending against screening for ASB, this practice is prevalent. We present outcomes from a quality-improvement intervention targeting patients undergoing cardiac artery bypass grafting surgery (CABG) at Massachusetts General Hospital, a tertiary-care hospital in Boston, Massachusetts, where preoperative testing checklists were modified to remove routine urinalysis and urine culture. This was a before-and-after intervention study.

**Methods:**

Prior to the intervention, screening for ASB was included in the preoperative check list for all patients undergoing CABG. We assessed the proportion of patients undergoing screening for ASB in the 6 months prior to and after the intervention. We estimated cost savings from averted laboratory analyses, and we evaluated changes in antibiotic prescriptions. We additionally examined the incidence of postoperative surgical-site infections (SSIs), central-line–associated bloodstream infections (CLABSIs), catheter-associated urinary tract infections (CAUTIs) and *Clostridioides difficile* infections (CDIs).

**Results:**

Comparing the pre- and postintervention periods, urinalyses decreased by 76.5% and urine cultures decreased by 87.0%, with an estimated cost savings of $8,090.38. There were 50% fewer antibiotic prescriptions for bacteriuria after the intervention.

**Conclusions:**

Removal of urinalysis and urine culture from preoperative checklists for cardiac surgery led to a statistically significant decrease in testing without an increase in SSIs, CLABSIs, CAUTIs, or CDI. Challenges identified included persistence of checklists in templated order sets in the electronic health record.

Preoperative checklists have been developed to standardize evaluation prior to surgeries and to reduce safety hazards and surgical complications while improving communication among operating room staff.^1,2^ These can be advantageous in that necessary tests are not forgotten prior to the procedure; however, low-value tests can be added to these checklists, and it becomes difficult to remove them once they are ingrained in practice. In a study assessing preoperative testing in an academic medical center, 52.9% of patients underwent at least 1 nonindicated laboratory test preoperatively.^3^ A qualitative study in 2011 found that persistence of ordering unnecessary tests is due to several reasons including practice tradition, desire to maximize information preoperatively, lack of knowledge of evidence and guidelines for appropriate testing, and concern surrounding delays if testing is not done.^4^

Historically, screening for and treating asymptomatic bacteriuria (ASB) preoperatively was considered necessary to prevent postoperative hematogenous seeding of foreign material from remote sources and associated surgical site infections (SSIs).^5^ Despite subsequent literature showing no relationship between treatment of preoperative ASB and postoperative infections, screening for ASB continues, with the vast majority of patients, up to 85%, screened preoperatively.^6–8^ Screening for ASB imposes costs on patients and insurers from testing, and when it results in unnecessary antibiotic prescriptions, it imposes additional costs associated with antibiotic prescriptions and risks of complications from antibiotics including *Clostridioides difficile* infection (CDI) and selecting for antimicrobial resistance.^9^

Several studies of both cardiac surgery and spinal cord surgeries have found no association with preoperative bacteriuria and postoperative infections.^7,10–12^ Despite recommendations against screening for ASB in nonpregnant adults from the US Preventative Services Task Force, or prior to nonurologic operations by the Infectious Disease Society of America, this practice has persisted.^13^ Cardiothoracic surgeries have had some of the highest screening rates for urine testing preoperatively.^8,9^

Avoiding testing for and treatment of ASB is an important opportunity for stewardship programs to focus on decreasing unnecessary prescribing of antibiotics.^14^ The Centers for Disease Control and Prevention (CDC) has identified collaboration with the microbiology laboratory, with a focus on diagnostic stewardship, as a core component to include within stewardship programs.^15^

We conducted a single-center study of an intervention to remove urinalysis and urine culture from a preoperative cardiac surgery checklist utilized for all patients undergoing cardiac artery bypass grafting (CABG). We assessed preoperative urinalyses performed, associated costs, antibiotic prescribing, and SSIs, catheter-associated bloodstream infections (CLABSIs), catheter-associated urinary tract infections (CAUTIs), and CDIs, prior to and after the decision to remove the testing from the checklist, and the associated educational campaign.

## Methods

### Setting

This study was conducted at Massachusetts General Hospital, a 1,063-bed tertiary-care facility in Boston, Massachusetts. There are 11 cardiac surgeons at this facility and approximately 1,454 cardiac surgeries are performed here annually.

### Urinalysis and urine-culture testing

The Massachusetts General Hospital Microbiology Laboratory does not use reflex urine- culture testing in which a urinalysis will reflex to a urine culture based on predefined criteria indicating infection. Therefore, patients often (but not always) received both a urinalysis and urine culture regardless of the appearance of the urinalysis. There is no decision support built into the electronic medical record at Massachusetts General Hospital requiring symptom attestation when ordering testing. Urine culture plating was performed using a 10-µL loop. Urine cultures were reported as no growth, rare bacteria (100 to <1,000 colony-forming units (CFU)/mL), few mixed bacteria or few identified organism (1,000 to <10,000 CFU/mL), or >100,000 CFU/mL of mixed bacteria or >100,000 CFU of an identified organism. According to our laboratory standard operating procedure, each colony on the plate is equivalent to 100 CFU/mL.

### Education session

On November 17, 2021, an education session was led by the study researchers (E.S.S., M.L.W., M.L.P., and A.R.L.) with cardiac surgery departmental leadership. In this hour-long session, information presented included existing national guidelines recommending against screening for ASB and the lack of a relationship between screening for and treating preoperative ASB and postoperative outcomes including infections. In this meeting, removal of screening urinalysis and urine culture from the preoperative checklist was discussed and agreed upon by all attendees.

### Study timeline and interventions

A fixed-timeframe study design was employed. The preintervention period spanned May 2021–November 2021; checklists were updated on November 18, 2021. The postintervention period, beginning after a wash-in period, was from December 2021 through May 2022. SSI surveillance extended for 90 days after the end of the study period. Interim analyses conducted in April 2022 and June of 2022 demonstrated that some providers were using versions of templated notes that contained checklists including preoperative urinalyses. These providers were directly notified by email regarding the removal of routine urine screening tests from the preoperative checklist.

### Assessed outcomes

Data were extracted from the electronic medical record using workbench reporting (Epic Systems, Verona, WI). Patients who underwent CABG and cardiac artery bypass grafting with chest and donor site incisions (CBGB) using National Healthcare Safety Network (NHSN) diagnostic coding criteria were identified.^16^ The primary outcome studied was collection of a preoperative urinalysis. Preoperative urinalyses were included if they were collected within 30 days of operative intervention.^9^ The time for collection for laboratory tests prior to surgery based on preoperative checklists ranged from 0 to 26 days in the preintervention arm (average, 6.4 days) and 0 to 27 days in the postintervention arm (average, 6.8 days). Manual chart review was conducted by 2 independent reviewers (J.H. and M.L.W.) to determine the reason for urinalysis and urine culture, results of testing, and antibiotics prescribed. SSIs, CLABSIs, CAUTIs, and CDIs within 30 days (CLABSI, CAUTI, CDI) or 90 days (SSI) of the procedure were assessed in accordance to NHSN criteria.^17^ ASB was defined as >100,000 CFU of a single organism in patients who did not otherwise have symptoms of an infection which were defined as fever, leukocytosis, suprapubic pain, increased urinary frequency, or shock identified by reviewing vital signs and notes.^14^ Patients with >100,000 CFU of mixed organisms were not included in our ASB definition. Antibiotic use was defined as either yes or no based on whether antibiotics were prescribed in response to the result of a urinalysis or urine culture.

### Laboratory costs

We used the Centers for Medicare and Medicaid Services clinical laboratory fee schedule from 2022 to estimate the cost of urinalysis and urine culture.^18^ An estimated cost of $29.98 per urinalysis was used (code 81007 or urinalysis for bacteria). An estimated cost of $8.09 per urine culture was used (code 87088, bacterial urine culture). We calculated the cost per reportable item for testing in terms of reagents used for testing. Utilizing facility data, the cost of a urine culture was $1.76; for a matrix-assisted laser desorption/ionization (MALDI) identification (Biomerieux, Durham, NC), the cost was $2.32, for susceptibility testing by the Vitek instrument (Biomerieux, Durham, NC), the cost was $5.35. The cost of a urinalysis was $0.67 and the cost of a urine sediment test was $2.67. We did not include the costs of instrumentation, labor, service, and overhead given that the volume of tests was not sufficient to reduce the number of technicians or instruments needed in the laboratory.

### Statistical analysis

After collection of the number of urinalyses, urine cultures, surgeries, and postoperative infections from the chart, statistical analyses were performed to compare the results before and after the intervention using Stata version 15.1 software (StataCorp, College Station, TX). Categorical variables were analyzed using the χ^2^ test if the expected number of events was >5 and the Fisher exact test if that number was <5. A 2-tailed *P* value of <.05 was considered significant.

## Results

### Patients

In total, 502 patients underwent 502 procedures between May 2021 and June 2022: 254 patients in the preintervention group and 248 patients in the postintervention group. Before the intervention, the average age was 67.7 years. After the intervention, the average age was 66.4 years. The preintervention group was composed of 46 women (18.1%) and 208 men (81.9%), and the postintervention group was compose of 35 women (14.2%) and 211 men (85.8%).

### Urinalysis and urine culture testing

**Figure 1.**
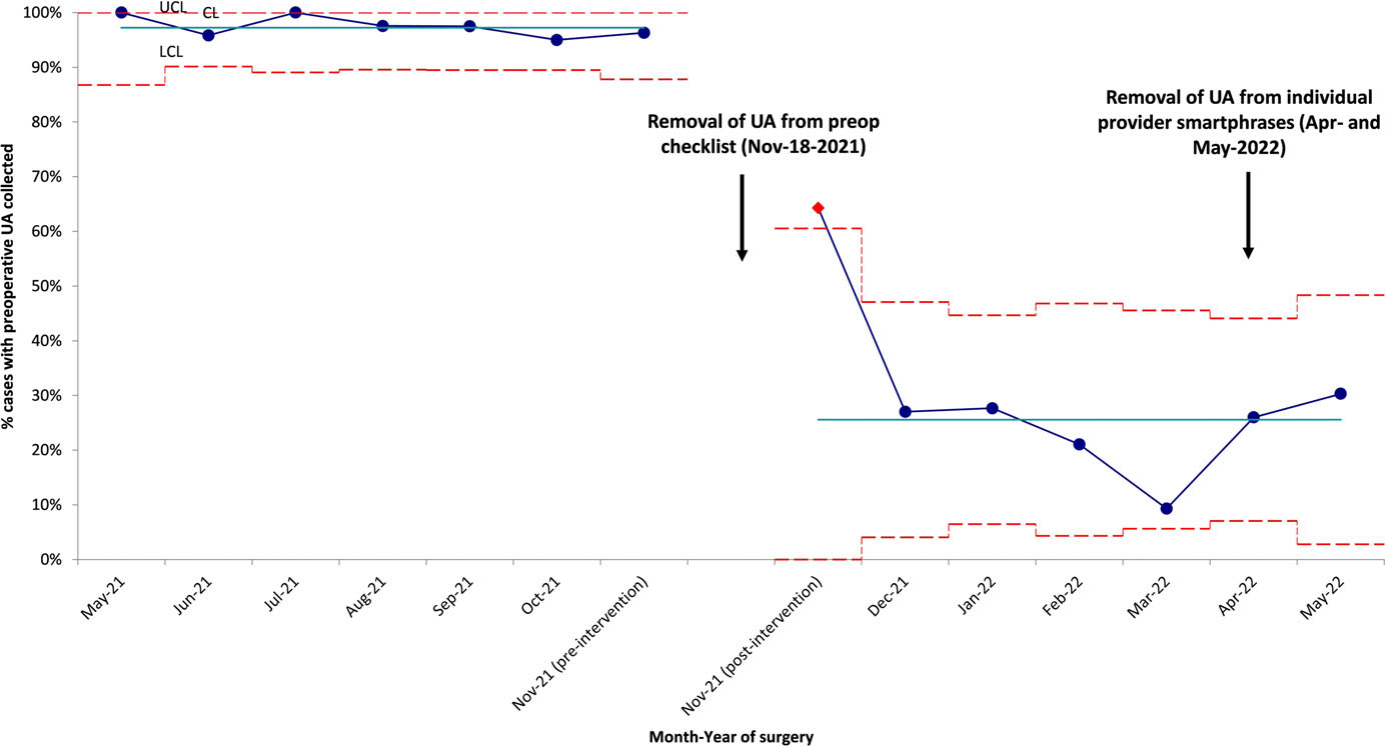
This 3σ p-chart describes the proportion of urinalysis (UA) collected from May 2021 to May 2022. Teal line presents the monthly average UA pre-intervention and post-intervention. Blue dotted line represents the percentage of UA collected preopreatively for each month. Dotted red lines represent the 3σ criteria describing data within 3 standard deviation from the mean. Upper control limit (UCL), central line (CL), and lower control limit (LCL) are set above, at, and below the mean respectively. Significant reduction in testing was seen after removal of UA from preoperative checklist.

Before the intervention, 247 (97.2%) of 254 patients had urinalyses performed (Fig. 1). Of these 254 patients, 76 (29.9%) had a urine sediment test and 236 (92.9%) had a urine culture. Among them, 34 MALDI identifications were made, and 16 Vitek susceptibilities were performed. Of the urinalyses, 44 (17.7%) were ordered by physicians, 174 (70.2%) were ordered by nurse practitioners (NPs), and 30 (12.1%) were ordered by physician assistants (PAs). Moreover, 113 (45.6%) of the tests were ordered based on outpatient clinic visits recommending the testing and 135 (54.4%) were ordered based on inpatient consultation recommending the testing through a checklist. 4 (3.5%) of 113 patients with testing recommended after outpatient appointments had >1 urinalysis performed, and 29 (21.5%) of 135 with testing recommended after inpatient consultation had >1 urinalysis performed. Of those who received a urinalysis, 12 patients did not have a urine culture. 6 patients did not receive a urinalysis or urine culture: 2 patients were oliguric, 2 patients were emergently brought to the operating room without standard preoperative laboratories, and 2 did not have listed reasons for lack of testing because it was recommended in the notes but was never performed. Of the patients with urine cultures, 9 patients were considered to have ASB. Of these patients, all testing was ordered due to the preoperative checklist. Of these 9 patients, 6 (66.7%) received antibiotics targeting their ASB. An additional 3 patients received antibiotics targeting bacteriuria that did not meet the CFU threshold for ASB (Table 1). For these patients, the urinalyses, urine culture, reason for ordering, and antibiotic treatment are shown in Table 1. All urine culture results are presented in Supplementary Table 1.

**Table 1.** Preintervention and Postintervention Urinalyses, Urine Cultures, and Antibiotics for Asymptomatic Bacteriuria

Urinalysis and Urine Sediment Result	Urine Culture Result	Reason for Testing	Antibiotic Prescribed	Sex	Age, Years
**Before the Intervention**					
3+ LE, 50–100 WBC	>100,000 CFU *Escherichia coli*	Checklist	Nitrofurantoin	M	53
1+ LE, positive nitrites, 10–20 WBC	>100,000 CFU *E. coli*	Checklist	Trimethoprim-sulfamethoxazole	M	72
1+ LE, <10 WBC	>100,000 CFU *E. coli*	Checklist	No antibiotics	M	72
3+ LE, positive nitrites, 50–100 WBC, positive squamous cells	>100,000 CFU *E. coli*, 1,000 to <10,000 CFU mixed bacteria	Checklist	Ciprofloxacin	F	76
Bland urinalysis, no sediment performed	>100,000 CFU *Enterococcus faecalis*, 100 to <1,000 CFU mixed bacteria	Checklist	Cefepime then ampicillin	M	81
3+ LE, >100 WBC, 3+ bacteria, 10–20 hyaline casts, 10–20 granular casts, CaOx, squamous cells	>100,000 CFU *Klebsiella pneumoniae* of 2 types	Checklist	Ceftriaxone	F	74
26 WBC	>100,000 CFU *Pseudomonas aeruginosa*	Checklist	No antibiotics	M	80
>100 WBC, + squamous cells	>100,000 CFU *E. coli*	Checklist	Nitrofurantoin	F	64
1+ LE, 10–20 WBC, + squamous cells	>100,000 CFU *E. faecalis*	Checklist	Cefepime	M	83
Bland urinalysis, no urinary sediment performed	10,000 to <100,000 CFU *E. faecalis*	Checklist	Ciprofloxacin, ampicillin-sulbactam	M	62
Negative nitrites, negative LE, 3–5 red blood cells, <10 WBC	10,000 to <100,000 *E. faecalis*, 1,000 to <10,000 mixed bacteria	Checklist	Ampicillin	M	67
Bland urinalysis, no urinary sediment performed	1,000 to <1,000 CFU *E. faecalis*	Checklist	Vancomycin	M	53
**After the Intervention**					
>100 WBC, + squamous cells	> 100,000 CFU *Corynebacterium* spp	Cough, no urinary symptoms	Ceftriaxone and doxycycline, ultimately deescalated to ampicillin-sulbactam	M	79
50–100 red blood cells, + squamous cells	>100,000 CFU *E. faecalis*, rare enteric gram-negative rods	Encephalopathy	Ceftriaxone and vancomycin, ultimately de-escalated to ampicillin	F	77
Bland urinalysis, no sediment performed	>100,000 CFU *E. faecalis*, rare mixed bacteria	Checklist	No antibiotics	F	71
50–100 WBC, + squamous cells	>100,000 CFU *K. pneumoniae*	WBC = 13, asymptomatic	Ciprofloxacin	M	77
Bland urinalysis, no sediment performed	10,000–100,000 CFU *E. coli*	Checklist	Ceftriaxone then levofloxacin	M	68

Note. LE, leukocyte esterase; WBC, white blood cell count; CFU, colony-forming units.

After the intervention, 58 (23.4%) of 248 patients had urinalyses performed, a reduction of 76.5% (*P* < .0005) (Fig. 1). For 29 (11.7%) of these 248 patients, urine sediment tests were performed. In 31 (12.5%) of 248 urine cultures, 6 had a MALDI identification and 5 Vitek susceptibilities performed. 36 (62.1%) of the urinalyses were ordered by physicians; 16 (27.6%) were ordered by NPs; and 6 (10.3%) were ordered by PAs. Furthermore, 9 (15.5%) of the tests were done on outpatients and 49 (84.5%) were done on inpatients. The reasons for testing after the intervention were more varied than before the intervention. For example, 19 of the patients had urinalyses drawn due to the use of an outdated preoperative checklist. Also, 2 of these patients had their preoperative visits prior to the intervention when preoperative testing was removed from the checklist. In addition, 13 patients did not have a listed reason for testing. Furthermore, 5 patients had symptoms: 2 had fever, 2 had shock, and 1 had increased urinary frequency. Also, 20 patients had other reasons for ordering urinalyses: encephalopathy, syncope, seizure, screening by primary care provider or oncology, systemic lupus erythematosus monitoring, or acute kidney injury. Another 6 patients had urinary testing ordered more than once (10.3%). Of the urinalyses after the intervention, 26 (45.6%) of 57 did not have an accompanying urine culture, and 4 patients had ASB (Table 1). Also, 3 (75%) of these patients received antibiotics targeting their ASB. Additionally, 1 patient received antibiotics targeting bacteriuria that did not meet the CFU threshold for ASB (Table 1).

### Secondary outcomes

Before the intervention, there were 10 SSIs, 1 CLABSI, and 2 CDIs. Of the patients with SSI, 3 of 10 urine cultures were obtained preoperatively with no growth, 5 had rare bacteria, 1 had few bacteria, and 1 had 50,000–100,000 CFU mixed bacteria. The SSIs were due to a variety of organisms (Supplementary Table 2). All patients received appropriate preoperative antibiotics with cefazolin within 1 hour of the surgical incision. The CLABSI was due to *Staphylococcus lugdunensis* and was diagnosed preoperatively. None of the patients with CDI or postoperative infection (SSIs or CLABSIs) received antibiotics prior to surgery targeting ASB. One received ceftriaxone for pneumonia, and another received a variety of antibiotics (meropenem, ciprofloxacin, and cefepime) for fever and pneumonia preoperatively. All patients receiving IV antibiotics were inpatients at the time of treatment.

After the intervention, there were 4 SSIs, 1 CLABSI, 1 CAUTI, and 3 CDIs. Of the patients with SSI, none had a preoperative urinalysis or urine culture. The SSIs were due to a variety of organisms (Supplementary Table 2), and all patients received appropriate preoperative antibiotics with cefazolin within 1 hour of the surgical incision. The CLABSI was due to *Serratia marcescens*; the patient had a preoperative urine culture that showed no growth. The CAUTI was due to *E. cloacae complex*; the patient had a preoperative urine culture with *Enterococcus faecalis* without antibiotics prescribed for ASB.

### Laboratory costs

After the intervention, there were 190 fewer urinalyses and 205 fewer urine cultures. The difference yielded an estimated cost savings to insurers of $5,696.20 from urinalyses and $1,658.45 from urine cultures, for a total of $7,354.65. There were $126.63, $124.49, $360.80, $64.96, and $58.85 in savings from averted urinalyses, urine sediments, urine cultures, MALDI identification, and Vitek susceptibility testing, respectively, for a total savings of $735.73. The cumulative savings was $8090.37.

## Discussion

We assessed the impact of removal of urinalysis and urine culture from a preoperative checklist in the cardiac surgery department of our hospital. Removal of unnecessary orders from a preoperative checklist after a short education session targeted at wasteful laboratory testing was a highly effective strategy to reduce unnecessary preoperative testing prior to CABG surgeries.

Despite education and stakeholder engagement, we observed a lag in implementation from some providers. This lag occurred most commonly in testing ordered by trainees who were not present during education sessions. The residents also less frequently perform the preoperative evaluations because most of these evaluations occur in the outpatient setting by a NP or PA. The evaluations performed by residents were all urgent evaluations in the inpatient setting. As a response to these findings, providers were all educated by email to ensure that they were aware of the change in screening policy.

In our analysis of reasons underlying ordering of urinalysis and urine cultures, we still found significant inappropriate use outside preoperative screening. Syncope, decompensated heart failure, and cough were all reasons for ordering urinalysis and urine culture despite lack of urinary symptoms. Panculturing as part of a workup for a variety of nonspecific symptoms has been previously addressed as an area to focus on urine culture stewardship.^19^ As individuals age, the likelihood of ASB increases, ranging from 1% to 8% in nonpregnant women to 15%–50% in residents of long-term care facilities.^14^ In our study, the rate of ASB was 3.8% in the preintervention population and 12.9% in the postintervention population (*P* = .696). This increase, though not statistically significant, may be due to a larger proportion of women having urine cultures performed after the intervention (8 of 31, 26%) than before the intervention (44 of 238, 18.5%). Women are known to have higher rates of ASB than men, and our ASB group comprised 33% women before the intervention and 50% women after the intervention.^13^

Additionally, many patients received multiple urinalyses and urine cultures, and these often occurred as patients transitioned between services. For example, a patient would have an initial test performed in the emergency department in a panculture approach and then a second culture performed for the checklist. Other patients were on antibiotics for a definitive nonurinary infection, and a urinalysis and urine culture were performed for the checklist, further increasing unnecessary costs of hospitalization. A future area of diagnostic stewardship that may address this would be an electronic medical record stop for repeated urine testing in a specific timeframe in the absence of new urinary symptoms.

Despite the limitations of our study, we were able to significantly reduce the amount of testing performed. Antibiotic use was lower, although not statistically significantly so (*P* = .11). Our study was not powered to detect a difference in antibiotic use. Postoperative infections did not increase, and no statistical difference was observed, although again the study was not powered to detect a change in postoperative infections. We detected a significant reduction in costs associated with urine testing in the postintervention period. In a different study of the economic cost of nonindicated preoperative urinalyses in all procedures, the total spending over a 16-month period in a single hospital facility was $48,675,408 with an additional $4,854,109 spent on antibiotics related to inappropriate urinalyses.^9^ In this study, we did not account for the additional costs of obtaining unnecessary tests to patients including lost productivity and inconvenience.

In summary, we have described a high-impact intervention to decrease unnecessary preoperative urinalyses and urine cultures prior to cardiac surgeries that was facilitated by an evidence-based education session regarding low-yield diagnostic tests in preoperative checklists. By removing urinalysis and urine culture from a preoperative checklist, we reduced unnecessary testing substantially.

## Supplementary material

For supplementary material accompanying this paper visit http://doi.org/10.1017/ash.2023.166. click here to view supplementary material
