# Supplementary material for: If you don’t test, they will not treat: Impact of stopping preoperative screening for asymptomatic bacteriuria
Source: Antimicrob Steward Healthc Epidemiol. 2023 May 26;3(1):e95. doi: 10.1017/ash.2023.166 (PMC10226188; doi:10.1017/ash.2023.166)
Supplement: Supplementary file 1 [file S2732494X23001663sup001.docx]

Supplemental Data

| **Supplemental Table 1: Summary of urine culture results** | | | |
| --- | --- | --- | --- |
| **Pre-intervention** | | **Post-intervention** | |
| Urine culture results | Number of patients | Urine culture results | Number of patients |
| No growth | 104 | No growth | 11 |
| >100,000 CFU *E. coli* | 3 | >100,000 CFU *Corynebacterium* | 1 |
| >100,000 CFU *E. coli*, 10,000 - < 100,000 *S. epidermidis* | 1 | >100,000 CFU *E. faecalis,* rare enteric GNRs | 1 |
| >100,000 CFU *E. coli*, 1000 - < 10,000 mixed bacteria | 1 | >100,000 CFU *E. faecalis,* rare mixed bacteria | 1 |
| >100,000 CFU *E. faecalis*, 100 - < 1000 mixed bacteria | 1 | >100,000 CFU *K. pneumoniae* | 1 |
| >100,000 CFU *E. faecalis*, 1000 - < 10,000 CFU GNR) | 1 | >100,000 CFU mixed organisms | 1 |
| >100,000 CFU *K. pneumoniae* | 1 | 10,000 - <100,000 CFU *E. coli* | 1 |
| >100,000 *P. aeruginosa*, 10,000 - 50,000 CFU mixed organisms | 1 | 10,000 - 50,000 CFU β-hemolytic Strep | 1 |
| >100,000 CFU mixed organisms | 1 | 10,000 - 50,000 CFU mixed organisms | 1 |
| 50,000 - 100,000 CFU mixed Gram-positive organisms | 1 | 1000 - < 10,000 probable *Enterococcus* | 1 |
| 10,000 - 50,000 CFU mixed organisms resembling cutaneous flora | 1 | 1000 - < 10,000 enteric GNRs, rare mixed bacteria | 1 |
| 10,000 - < 100,000 CFU *E. faecalis* | 2 | 1000 - < 10,000 mixed bacteria | 5 |
| 10,000 - 100,000 CFU *E. faecalis*, 10,000 - < 100,000 CFU Strep mitis/oralis | 1 | 100 - < 1000 mixed bacteria | 5 |
| 10,000 - < 100,000 *E. faecalis*, 1000 - < 10,000 mixed bacteria | 1 |  |  |
| 10,000 - < 100,000 CFU Strep mitis/oralis | 1 |  |  |
| 10,000 - < 100,000 CFU *S. agalactiae*, 100 - < 1000 CFU mixed bacteria | 1 |  |  |
| 10,000 - < 100,000 CFU *P. aeruginosa* of two types | 1 |  |  |
| 10,000 - < 100,000 CFU mixed bacteria | 2 |  |  |
| 1000 - < 10,000 CFU *E. faecalis* | 7 |  |  |
| 1000 - < 10,000 CFU *E. faecalis*, 100 - < 1000 CFU GNRs | 2 |  |  |
| 1000 - < 10,000 CFU *E. faecalis*, and 1000 - < 10,000 CFU mixed bacteria | 1 |  |  |
| 1000 - < 10,000 CFU Enterococcus, 10,000 -< 100,000 CFU mixed bacteria | 1 |  |  |
| 1000 - < 10,000 CFU Enterococcus, 100 - < 1000 CFU mixed bacteria | 1 |  |  |
| 1000 - < 10,000 CFU *E. coli* 100 - < 1000 CFU *E. faecalis* | 1 |  |  |
| 1000 - < 10,000 CFU mixed bacteria | 7 |  |  |
| 1000 - < 10,000 CFU yeast, 100 - < 1000 CFU GNRs of two types | 1 |  |  |
| 1000 - < 10,000 CFU yeast, 100 - < 1000 CFU Enterococcus | 1 |  |  |
| 1000 - < 10,000 CFU enteric GNRs, 100 - < 1000 CFU mixed bacteria | 1 |  |  |
| 100 - < 1000 CFU enteric GNRs, 100 - < 1000 CFU GNRs | 1 |  |  |
| 1000 - < 10,000 CFU group B strep, 1000 - < 10,000 CFU mixed bacteria | 1 |  |  |
| 1000 - < 10,000 mixed bacteria | 8 |  |  |
| 1000 - < 10,000 CFU GNRs | 4 |  |  |
| 1000 - < 10,000 CFU GNRs, 10,000 - < 100,000 CFU mixed bacteria | 1 |  |  |
| 1000 - < 10,000 CFU *M. morganii*, 1000 - < 10,000 CFU *S. aureus* | 1 |  |  |
| 1000 - < 10,000 CFU β-hemolytic Strep, 1000 - < 10,000 mixed bacteria | 1 |  |  |
| 1000 - < 10,000 CFU mixed bacteria | 15 |  |  |
| 100 - < 1000 CFU Enterococcus | 5 |  |  |
| 100 - < 1000 CFU GNRs | 5 |  |  |
| 100 - < 1000 CFU mixed bacteria | 35 |  |  |
| 100 - < 1000 CFU Enterococcus | 2 |  |  |
| 100 - < 1000 CFU *S. epidermidis* | 2 |  |  |
| 100 - < 1000 CFU *Staph spp* | 3 |  |  |
| 100 - < 1000 CFU alpha hemolytic Strep | 1 |  |  |
| 100 - < 1000 CFU Lactobacillus spp | 1 |  |  |

| **Supplemental Table 2: Summary of postoperative infections pre- and post-intervention** | | | | | | | | | |
| --- | --- | --- | --- | --- | --- | --- | --- | --- | --- |
| **Pre-intervention** | | | | | **Post-intervention** | | | | |
| **Type of infection** | **SSI** | **CDI** | **CRBSI** | **CAUTI** | | **SSI** | **CDI** | **CRBSI** | **CAUTI** |
| **Number of infections** | 10 | 2 | 1 | 0 | | 4 | 3 | 1 | 1 |
| Organism responsible | *Serratia marcescens* (1*), Morganella morganii* (1), *Pseudomonas aeruginosa* (2), methicillin-susceptible *S. aureus* (1), *Klebsiella pneumoniae* and *Haemophilus parainfluenzae* (1), *E. coli* (1), and no growth (3) |  | *S. lugdunensis* |  | | *P. aeruginosa* (2), *Enterobacter cloacae complex* (1), no organism (1) |  | *S. marcescens* | *E. cloacae complex* |
